# Study of the Urinary TGF-β1 Profile in Diabetic Nephropathy: A Single-Center Experience From India

**DOI:** 10.7759/cureus.45102

**Published:** 2023-09-12

**Authors:** Akshay R Kulkarni, Charan B Bale, Pavan S Wakhare, Nilesh S Shinde, Abhijit S Chavan, Tushar A Dighe, Atul D Sajgure

**Affiliations:** 1 Nephrology, Dr. D. Y. Patil Medical College, Hospital & Research Centre, Dr. D. Y. Patil Vidyapeeth, Pune, IND

**Keywords:** india, proteinuria, chronic kidney disease (ckd), tgf-β1, diabetic nephropathy (dn)

## Abstract

Background

Diabetic nephropathy is one of the important causes of end-stage kidney disease (ESKD). Of the various cytokines playing a role in the pathogenesis of diabetic nephropathy, transforming growth factor beta-1 (TGF-β1) is an important one. Its major role is to mediate extracellular matrix deposition. Increased renal expression of TGF-β1 is found in diabetic nephropathy and its urinary excretion can serve as a useful marker of outcomes.

Material and methods

A prospective observational study was conducted, which included 10 cases of diabetic nephropathy in group A with age ≥ 18 years and a urinary protein creatinine ratio (UPCR) value of > 0.5 mg/mg and 10 healthy controls in group B. Patients with active urinary tract infection, chronic kidney disease (CKD) stage Vd patients on maintenance hemodialysis, and renal transplant recipients were excluded from the study. Urinary TGF-β1 level estimation in a 24-hour urine sample, 24-hour urine protein, and other baseline laboratory investigations were done.

Results

In diabetic nephropathy cases (group A), the mean value of urinary TGF-β1 levels was 88.33± 12.44 ng/24 hours. In the control group (group B), the mean value of urinary TGF-β1 was 29.03 ± 3.23 ng/24 hours. Urinary TGF-β1 levels were significantly elevated in group A as compared to group B (p<0.001). There was no significant correlation between urinary TGF-β1 levels and estimated glomerular filtration rate (eGFR) (r=0.376, p= 0.285) as well as the urinary TGF-β1 levels and 24-hour urine protein levels (p = 0.334, r = 0.341) in diabetic nephropathy cases. Glycosylated hemoglobin (HbA1c) levels didn’t correlate with the urinary TGF-β1 levels (r = -0.265, p = 0.46).

Conclusion

The urinary TGF-β1 levels were significantly elevated in diabetic nephropathy patients as compared to healthy controls. There was no significant correlation between urinary TGF-β1 levels and proteinuria, eGFR, or HbA1c levels in diabetic nephropathy patients.

## Introduction

India is the world capital of diabetes and the prevalence of diabetes in India is around 8.9% [1]. In patients with diabetes, diabetic nephropathy (DN) is the most important cause of end-stage kidney disease (ESKD). Early diabetic kidney disease is associated with low-level albuminuria of 30-300 mg per day, i.e. microalbuminuria, which later progresses to macroalbuminuria. It is a marker of underlying kidney disease, mediates progressive renal dysfunction, and is a powerful and independent risk factor for cardiovascular disease development [2,3]. Excessive production of extracellular matrix (ECM) leads to renal fibrosis. This is the hallmark of progressive chronic kidney disease (CKD). A key mediator in the pathogenesis of DN is transforming growth factor beta-1 (TGF-β1). which is considered to be a potent profibrotic mediator in this process [4-6]. TGF-β1 is a cytokine with multiple functions. It is recognized to control a wide range of cellular processes like growth, wound repair, apoptosis, differentiation, and fibrosis [7]. Increased levels of TGF-β1 were found to be associated with severe glomerulonephritis and glomerulosclerosis [8]. Even though the pro-inflammatory role of TGF-β1 is well-established, it also has some anti-inflammatory effects [9]. The targeted deletion of the TGF-β1 gene in mice resulting in a multifocal inflammatory disease was the supporting evidence in this case [10]. Several studies have found that urinary TGF-β1 levels were significantly elevated in diabetic nephropathy patients, but none of these studies were conducted in India. With this background, the aim of our study was to assess the profile of urinary TGF-β1 in diabetic nephropathy patients.

## Materials and methods

It was a prospective observational study in western India over a period of 18 months. Before commencing the study, approval was taken from the institutional ethics committee (Dr. D. Y. Patil Vidyapeeth, Pune, India, protocol no. IESC/S.SP/2020/02). We also obtained informed and written consent from all the study participants. The participants were enrolled under two arms: 10 cases of diabetic nephropathy (group A) and 10 healthy controls (group B). Inclusion criteria for cases were age ≥ 18 years, urinary protein creatinine ratio (UPCR) value of > 0.5 mg/mg, and those willing to give informed consent for participation in the study. Patients with age less than 18 years, presence of active urinary tract infection, CKD stage Vd patients on maintenance hemodialysis, and renal transplant recipients were excluded from the study. Patients fulfilling the selection criteria were briefed about the nature of the study. Participants were interviewed to obtain information regarding demographic characteristics such as age and sex, history of diabetes, other co-morbidities, and presenting complaints. Study participants underwent a thorough clinical examination and findings were recorded in a predesigned proforma.

Urinary TGF-β1 levels

All the study participants collected 24-hour urine samples. The total volume of the sample was noted. Twenty (20) µl of urine was analyzed for the presence of TGF-β1 using multicolor flow cytometry with the MACSQuant® Analyzer 10 Flow Cytometer (Miltenyi Biotec, Bergisch Gladbach, Germany). In 1.5 ml tubes, 20 µl of urine was admixed with 20 µl of cytometric array capture beads. At room temperature, these samples were incubated for two hours. After incubation, 50 µl phosphate-buffered saline (PBS) was added. It was centrifuged for 5 minutes at 5000 rpm, and a volume of 70 µl was removed. In this sample, 20 µl detection beads were added. These samples were incubated for another two hours. After incubation, 160 µl buffer was added and samples were analyzed using multicolor flow cytometry. Every sample was run in triplicates. The average of the three readings was taken.

Spot urinary protein estimation was done using the pyrogallol red method, performed using Dimension EXL 200 Integrated Chemistry System (Siemens Healthcare Diagnostics Inc., Malvern, PA). Spot urinary creatinine estimation was performed by the alkaline picrate kinetic method using Dimension EXL 200 Integrated Chemistry System. The urine protein: creatinine ratio was calculated as -Urine protein: creatinine ratio (mg/mg) = urine protein (mg/dl)/ urine creatinine (mg/dl). Complete blood count (CBC) was obtained using a 3 ml EDTA blood sample with the DxH 800 Hematology Analyzer (Beckman Coulter, Inc. USA. Urine analysis was done on freshly voided, clean catch, mid-stream urine sample, using the UC-3500 fully automated urine chemistry analyzer (Sysmex Corporation, Kobe, Japan). A 24-hour urine protein (mg/24 hours) estimation was done by the pyrogallol red method using the Dimension EXL 200 Integrated Chemistry System. A 24-hour urine creatinine (mg/24 hours) estimation was done by the alkaline picrate kinetic method using the Dimension EXL 200 Integrated Chemistry System. The estimated glomerular filtration rate (eGFR) was calculated using the CKD-EPI 2021 (Chronic Kidney Disease Epidemiology Collaboration) equation [11]. All other routine laboratory investigations were done as per standard methods and protocol.

Statistical analysis

Data were collected using a semi-structured questionnaire. Data were entered in Microsoft Excel 2019 (Microsoft Corporation, Redmond, WA). Non-continuous data were represented in frequencies and percentages and continuous data were expressed as mean and standard deviation. For comparing non-continuous variables, the chi-square test was used, and the student’s t-test was used for comparison of continuous variables. Quantitative variables were correlated using Pearson’s correlation. IBM SPSS Statistics for Windows, version 22.0 (Armonk, NY: IBM Corp.) was used to apply appropriate statistical tests for data analysis. Statistical significance was considered at p < 0.05 at a 95% confidence interval.

## Results

Demographic and clinical characteristics

In the diabetic nephropathy group (group A), the mean age of the study participants was 51.30 ± 10.61 years, and in the control group (group B), it was 33.90 ± 10.82 years. There were eight (80%) males and two (20%) females in group A and six (60%) males and four (40%) females in group B. There was no significant difference between the mean body mass index (BMI) value of the two groups (group A 25.44 ± 3.26 kg/m^2^ and group B 25.88 ± 3.07 kg/m^2^, p=0.759). There was a significant difference in the mean value of systolic BP between the two groups (132.80 ± 12.69 and 121.90± 6.12 mmHg, respectively, p<0.05). The mean value of 24-hour urine output in group A was 1565.00 ± 131.34 ml and in group B, it was 1705.00 ± 258.68 ml. Table 1 compares the clinical and laboratory parameters of group A and group B.

**Table 1:** Comparison of the clinical and laboratory parameters of group A and group B BMI = body mass index, eGFR = estimated glomerular filtration rate, Hb = haemoglobin, TGF-β1 = transforming growth factor beta-1, UPCR = urinary protein creatinine ratio *= statistically significant

Parameter	Group A (Mean ± SD)	Group B (Mean ± SD)	P value
Age (years)	51.30± 10.61	33.90± 10.82	0.002*
Gender (Male/Female)	8/2	6/4	-
Height (meter)	1.66± 0.07	1.65± 0.07	0.622
Weight (Kg)	70.60± 11.97	70.20± 10.87	0.939
BMI (kg/m^2^)	25.44± 3.26	25.88± 3.07	0.759
Systolic BP (mmHg)	132.80± 12.69	121.90± 6.12	0.025*
Diastolic BP (mmHg)	83.40± 13.17	80.70± 5.74	0.560
Hb (gm/dl)	10.62± 1.62	13.30± 1.44	0.001*
Serum Albumin (gm/dl)	2.58± 0.34	3.30± 0.19	<0.001*
Blood urea level ( mg/dl)	72.40± 34.25	42.00± 4.76	0.012*
Serum Creatinine (mg/dl)	2.72± 1.87	0.86± 0.18	0.006*
eGFR (ml/min/1.73 m^2^)	41.90± 30.49	104.80± 21.99	<0.001*
UPCR (mg/mg)	5.41± 3.61	0.14± 0.06	<0.001*
24 hours urine protein (mg)	5763.10±2801.43	133.60± 21.01	<0.001*
24 hours urine creatinine (mg)	1617.20± 384.83	1244.90± 192.26	0.014*
Urinary TGF-β1 level (ng/24hr)	88.33± 12.44	29.03± 3.23	<0.001*

Urinary TGF-β1 level

**Figure 1:**
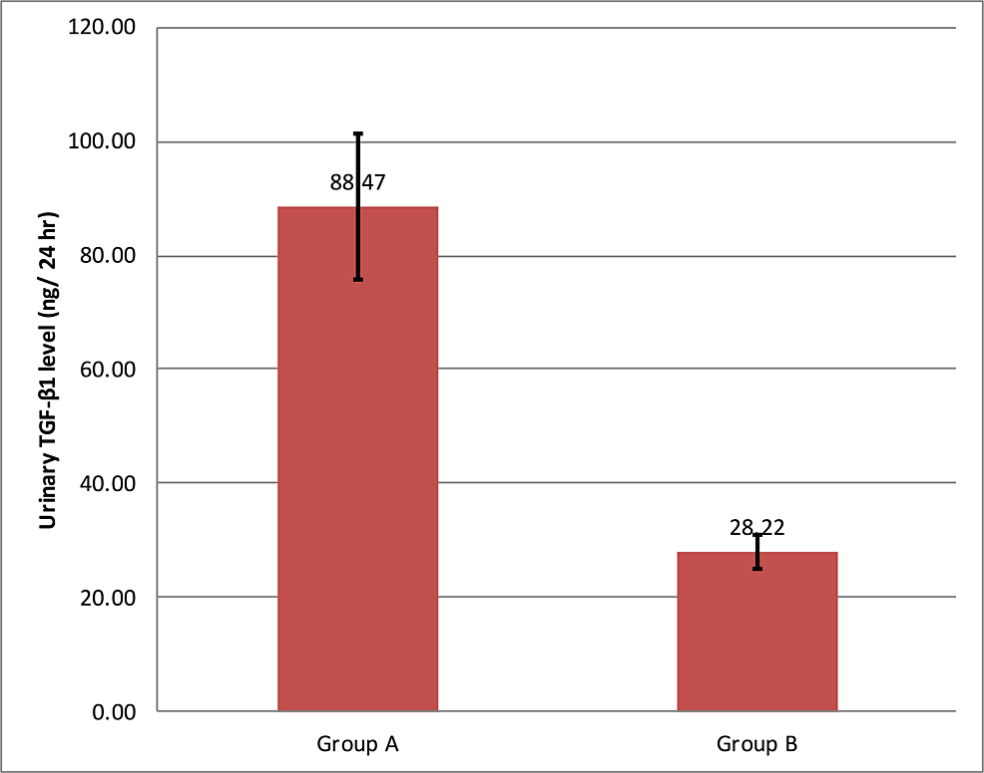
Comparison of mean urinary TGF-β1 levels of groups A and B TGF-β1 = transforming growth factor beta-1

In diabetic nephropathy cases (group A), the mean value of urinary TGF-β1 levels was 88.33 ± 12.44 ng/24 hours. In the control group (group B), the mean value of urinary TGF-β1 was 29.03 ± 3.23 ng/24 hours. Urinary TGF-β1 levels were significantly elevated in group A compared to group B (p<0.001). Figure 1 shows a comparison of the mean urinary TGF-β1 levels of groups A and B.

TGF-β1 levels and eGFR

**Figure 2:**
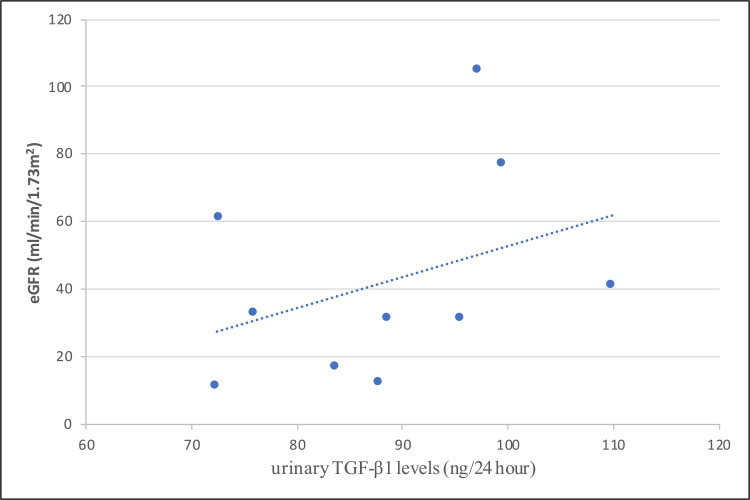
Correlation between urinary TGF-β1 levels and eGFR in group A eGFR = estimated glomerular filtration rate, TGF-β1 = transforming growth factor beta-1

There was no significant correlation between urinary TGF-β1 levels and eGFR (r =0.376, p=0.285) among diabetic nephropathy patients, as shown in Figure 2.

TGF-β1 levels and proteinuria

**Figure 3:**
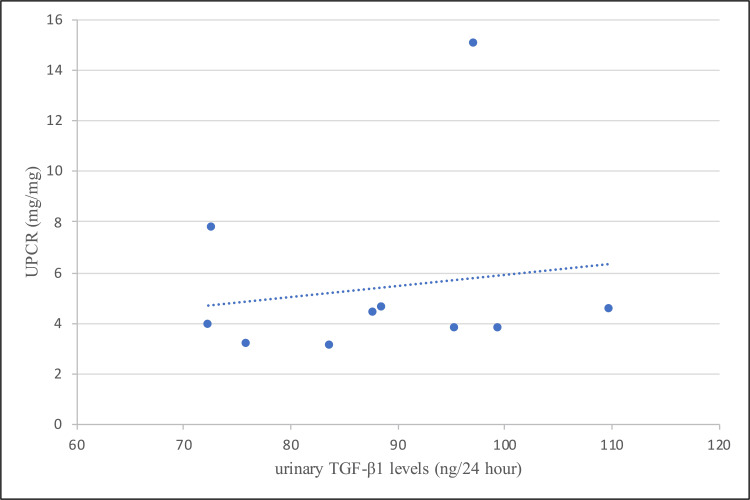
Correlation between urinary TGF-β1 level and UPCR in group A TGF-β1= transforming growth factor beta-1, UPCR= urinary protein creatinine ratio.

The mean value of 24-hour urine protein was 5763.10 ± 2801.43 mg in group A and 133.60 ± 21.01 mg in group B. There was no significant correlation between urinary TGF-β1 levels and 24-hour urine protein level (p = 0.334, r = 0.341, Pearson correlation) in diabetic nephropathy patients. Group A had a mean UPCR of 5.41 ± 3.61 mg/mg and that of group B was 0.14 ± 0.06 mg/mg. There was no significant correlation between urinary TGF-β1 levels and UPCR among diabetic nephropathy (group A) cases (r = 0.154, p = 0.672), as seen in Figure 3.

TGF-β1 levels and HbA1c

The mean value of HbA1c in group A was 8.09 ± 0.88%. There was no significant correlation between urinary TGF-β1 levels and HbA1c in diabetic nephropathy patients (Pearson correlation, r=-0.265,p = 0.46).

## Discussion

In the human kidney, three isoforms of TGF-β are expressed: TGF-β1, TGF-β2, and TGF-β3 [12]. Of these three, the predominant one is TGF-β1. In the adult human kidney, TGF-β2 and TGF-β3 are mainly expressed in podocytes and TGF-β1 in tubules. However, TGF-β1 was detected in mesangial cells in diabetic nephropathy and IgA nephropathy cases [13,14]. Its major role in diabetic nephropathy has always been an area of interest. Various studies have been done to assess the profile of urinary TGF-β1.

Our study found urinary TGF-β1 levels to be significantly high in diabetic nephropathy patients compared to healthy controls (p< 0.001). The finding of significantly raised urinary TGF-β1 levels was similar to studies done in diabetic nephropathy patients by Sato H et al., Sauriasari and Pratiwi, and Shaker YM et al. [15-17].

The molecular weight of TGF-β1 is around 25 kD, almost one-third that of albumin. The glomerular basement membrane can permeate its passage. Sato H et al. performed an immunofluorescent study in patients with type 2 DM and showed that TGF- β1 is localized to the areas of mesangial expansion [15]. In addition, serum TGF-β1 levels did not correlate with urinary TGF-β1 concentrations. The overproduction of TGF-β1 in glomeruli is the main contributor to the increased urinary excretion of TGF-β1, as per these findings. The concentrations of parameters of tubular dysfunction like serum creatinine levels did not correlate with urinary TGF-β1 in the study done by Sato H et al. [15]. This discounted the possibility of TGF-β1 secretion in urine due to the damage of protein reabsorption by tubular epithelial cells. Our study findings were similar to Sato H et al. and there was no correlation of urinary TGF-β1 levels with serum creatinine and eGFR in group A [15].

Similar to our finding in diabetic nephropathy patients, Sauriasari and Pratiwi found no correlation of urinary TGF-β1 levels with proteinuria measured as urinary albumin creatinine ratio (UACR) [16]. On the contrary, Shaker YM et al.'s study found a high positive correlation of urinary TGF-β1 with total urine proteins in the macro and microalbuminuria groups among diabetic nephropathy patients [17]. These findings suggest that urinary TGF-β1 levels reflected renal production rather than urinary protein excretion. In the study done by Sauriasari and Pratiwi, the concentration of urinary TGF-β1 was increased in albuminuric patients, but it was not statistically significant [16]. Most of the participants in their study received antihypertensive medications, including angiotensin receptor blockers (ARBs) or angiotensin-converting enzyme (ACE) inhibitors. These medications would have potentially decreased the level of urinary TGF-β1. In our study, 80% of patients from group A received either ARBs or ACE inhibitors. This would have probably led to the absence of a correlation between TGF-β1 and UPCR or eGFR in our study.

In diabetic nephropathy patients, the HbA1c level didn’t correlate with the urinary TGF-β1 level in our study. Sato H et al. divided diabetic nephropathy patients based on HbA1c levels into two groups [15]. Patients with HbA1c >6.5% had significantly higher levels of urinary TGF-β1 compared to those with HbA1c <6.5% (p < 0.05). Shankland et al. have demonstrated that during the early phase of renal hypertrophy, TGF-β1 expression increases in glomeruli [18]. In diabetic rats, this increased TGF-β1 expression was found to be reduced after treatment with insulin and normalization of blood sugar levels. These reports pointed toward a close relationship between TGF-β1 production in the glomeruli and blood glucose levels. The difference in our study findings can be possibly due to the small sample size of diabetic nephropathy cases as compared to Sato H et al. (n=10 vs. n=57) [15].

Our study had the limitation of a smaller sample size and short duration of follow-up. A larger sample size and a longer follow-up might have established a significant correlation between variables.

## Conclusions

Our study findings showed that urinary TGF-β1 levels were significantly elevated in diabetic nephropathy patients compared to healthy controls. There was no significant correlation between urinary TGF-β1 levels and proteinuria, serum creatinine level, or eGFR. Among diabetic nephropathy cases, no correlation was observed between HbA1c levels and urinary TGF-β1 levels. The study findings difference can be attributed to a small sample size and possibly to differences in ethnicity and genetic composition.
